# Social and Cultural Barriers and Facilitators Affecting Mothers’ Dietary Practices in Eastern Ethiopia: A Qualitative Study

**DOI:** 10.1016/j.cdnut.2025.107602

**Published:** 2025-11-14

**Authors:** Ketema Degefa, Luisa Schneider, Freek Colombijn, Kedir Teji Roba

**Affiliations:** 1 Faculty of Social Sciences and Humanities, Department of Social and Cultural Anthropology, Vrije Universiteit Amsterdam, Amsterdam, The Netherlands; 2 Department of Public Health, College of Health and Medical Sciences, Haramaya University, Harar, Ethiopia

**Keywords:** dietary practices, pregnant females, dietary self-restraint, barriers, facilitators

## Abstract

**Background:**

Inadequate dietary practices during pregnancy and breastfeeding, adversely affecting the health of both mothers and their children, are a serious public health challenge in Ethiopia. Analyzing barriers and facilitators will help take appropriate steps to facilitate change and enable better dietary practices.

**Objectives:**

The aim of this study is to analyze barriers to and facilitators of adequate dietary practices in Eastern Ethiopia, and to help health workers recognize existing adequate dietary practices and implement effective nutritional interventions.

**Methods:**

Qualitative research was conducted with in-depth interviews, focus group discussions, and participant observation. The interviews and focus group discussions involved 112 participants: 27 pregnant females, 30 breastfeeding females, 49 health workers, 3 traditional birth attendants, and 3 religious leaders (sheikhs).

**Results:**

The study identified the social and cultural barriers and facilitators of dietary practices. Barriers to adequate dietary practices included inadequate family income, the sale of self-produced healthy food items to purchase less healthy packed food, prioritizing family members, dietary self-restraint, khat use, and religious fasting practices. Facilitators of dietary practices included positive religious leaders’ views, cooking demonstrations, and pregnancy conferences (public health forums for pregnant females in Ethiopia).

**Conclusions:**

Understanding the social and cultural barriers and facilitators of dietary practices can help health workers effectively implement existing interventions to support pregnant and breastfeeding females. However, the females had partly different priorities than the health workers in making dietary choices and kept more factors in mind than nutritional considerations. Cooking demonstrations and pregnancy conferences were, alongside regular health extension sessions, effective moments to encourage pregnant females to increase dietary diversity, because there the females had the opportunity to exchange experiences.

## Introduction

Adequate dietary practices during pregnancy have a positive effect on mothers, fetuses, and newborn infants [1,2]. Dietary practices can be defined as observable actions or behaviors related to dietary habits [3]. Healthy eating practices among pregnant females support fetal growth, enhance fetal brain development, and prevent complications in both the mother and the newborn [4,5]. After birth, healthy dietary practices remain essential for breastfeeding mothers and their newborn children and may also reduce risk of chronic diseases later in life [6,7]. Conversely, several studies have reported the health consequences of inadequate dietary practices. An inadequately balanced diet for a pregnant female can result in premature birth and low birth weight, affecting the child’s growth and health [[8], [9], [10]]. Inadequate nutrition during pregnancy causes anemia, preterm delivery, and intrauterine growth restriction [11,12].

Ethiopia is one of the sub-Saharan countries with the highest malnutrition problems. Knowledge, accessibility, economic affordability, and cultural norms constitute barriers to or facilitators of dietary practices among pregnant and breastfeeding females in Ethiopia [8,[11], [12], [13]]. Knowledge about dietary diversity (usually measured as the number of different food groups consumed over a certain period) and nutrient adequacy (diets that meet requirements of energy and essential nutrients) is positively associated with adequate nutrition [14,15]. Knowledge is associated with general educational status [16], but also with targeted counseling. Pregnant females who received dietary counseling during antenatal care have developed more adequate dietary practices [17]. Financial constraints are a barrier to dietary diversity among pregnant females [17]. However, in sub-Saharan countries, including Ethiopia, higher economic status and educational level do not necessarily ensure improved dietary diversity [[18], [19], [20]]. Cultural norms play a role in various ways and include customs about the allocation of food between family members and the degree of maternal decision-making autonomy [16].

The availability of health systems at the community level is an enabler in promoting the importance of a balanced diet during pregnancy, though progress is slow. The Ethiopian government introduced the Community Health Extension Program in 2003, consisting of 17 packages, which include nutrition intervention activities [21]. Health extension workers are responsible for promoting dietary diversity, in close cooperation with village leaders, traditional birth attendants, religious leaders, and other community leaders. The health extension workers undertake nutritional counseling of pregnant females during their visits to health posts and outreach activities in the communities, including nutritional education and cooking demonstrations for pregnant females and breastfeeding mothers. However, the intervention activities are hindered by institutional constraints: the workload of health workers [22,23], poor living conditions that stimulate health workers to seek other jobs [21], irregular closing times [24], and a shortage of iron-folate and deworming tablets [25]. The success of such national health extension programs is also contingent on their adaptation to local cultures and social systems [26,27]. Lifestyles in rural Ethiopia are not uniform, and understanding local variation is essential for aligning national nutrition intervention activities to local cultural practices and social systems. To fully understand what promotes or obstructs inadequate dietary practices locally, empirical studies are necessary [13].

Our study focuses on social and cultural facilitators of and barriers to food diversity in 1 region of Eastern Ethiopia, the district of Kersa. A better understanding of local social and cultural practices will help health extension workers to recognize positive existing practices and intervene in a socially effective manner where required. Cultural practices differ from place to place and, therefore, cannot be assumed but must be studied empirically.

These cultural practices can influence dietary practices in several ways. Previous studies have examined food taboos among pregnant females [28,29], but in our study we have used the broader term dietary self-restraint to explore how pregnant females refrain from consuming some food items. Due to cultural taboo, certain foods, such as linseed, pumpkin, and chickpeas, are avoided during pregnancy to the extent that they can reduce a pregnant female’s food intake [30]. Religion can be considered as 1 cultural domain that influences the dietary practices of pregnant and breastfeeding females. Islam is the majority religion in Eastern Ethiopia, and during Ramadan, the intake of all food and fluids is prohibited during daytime. Although this is not strictly required from pregnant and breastfeeding females, many fast as well during Ramadan [31,32]. The second largest religious group in Eastern Ethiopia, belonging to the Orthodox Tewahedo Church, specifically abstains from animal dairy products during fasting. Also, pregnant females follow this religious fasting, which includes milk, eggs, meat, and butter [32].

Although the above-cited studies have identified factors that influence the dietary practices of pregnant females, they pay less attention to the beliefs the pregnant females themselves have about the relationship between dietary practices and the perinatal health of mother and child. For instance, earlier research in Ethiopia found that some females believe in a self-chosen restriction of protein and vitamin-rich foods because they are concerned that consuming such foods may lead to having a larger infant. They think that a larger infant could complicate delivery, and increase the likelihood of a cesarean section or their own death at delivery [33]. Our research focuses on barriers and facilitators of dietary practices and females’ perceptions and contrasts their views with the opinions of health workers. The central research question is: What social and cultural barriers and facilitators affect mothers’ dietary practices in Eastern Ethiopia, particularly their beliefs surrounding nutritional restrictions?

## Methods

### Study setting

The study was conducted in Eastern Ethiopia, at the Health Demographic Surveillance Site (HDSS) of the district of Kersa. HDSS are standard sites used for surveys, where longitudinal data are collected. Kersa HDSS covers 24 of the total of 38 subdistricts of the district of Kersa. The national census of 2007 reported a total population of 170,816 in this district, consisting of 86,134 males and 84,682 females [34]. Khat serves as the primary source of household income and is also exported to neighboring countries, including Djibouti and Somalia [21,35]. Wheat, barley, and vegetables are the dominant food crops in the highlands, and sorghum, maize, and potatoes are common in the midland and lowland regions [36]. This study found that animal products, including eggs, milk, and meat from goats and cows, are primarily produced for sale and not for their own consumption.

The institutional ethics committee of the College of Health and Medical Sciences, Haramaya University (IHRERC/047/2023) approved this protocol. Additionally, the Research Ethics Review Committee (RERC) of the Faculty of Social Sciences, Vrije Universiteit Amsterdam, approved the protocol (RERC/23-01-3). Written informed consent was obtained from most study participants by signing (or thump-printing in case they were illiterate) on the consent form. Study objectives and consent were explained to the study participants. During the data collection, study participants were informed that there was no risk of participating in the study. Participants were informed that their data would be kept private. We securely protect participants’ privacy. The audio recorders were securely stored in locked cabinets, and all databases and computers were protected with passwords.

### Study design and period

A qualitative ethnographic study design was employed; for triangulation, we conducted in-depth interviews, held focus group discussions, and conducted participant observation during moments that pregnant or breastfeeding mothers interacted with each other and health workers at extension activities. The ethnographic methods allowed an open discussion of sensitive topics, the potential discovery of unexpected findings not covered in surveys, and an analysis of discrepancies between what people said they did and what they actually did. Ethnographic research yields detailed and comprehensive accounts of various social phenomena, including behaviors, social interactions, and beliefs of the study population [37]. The study was conducted between April and June 2023 and again from February to March 2024. Fieldwork was conducted in 2 periods for practical reasons and had the advantage of a round of analysis before fieldwork was resumed in 2024. The policies for treating malnutrition at the health centers did not change between these 2 periods, but taken together, the periods cover both preharvest and postharvest seasons.

### Participants and data collection

We used purposive sampling techniques for the selection of 112 participants in total. In-depth interviews were held with 16 pregnant females, 21 breastfeeding females, 6 health workers, 3 traditional birth attendants, and 3 religious leaders, called sheikhs. We held 7 focus group discussions with in total 11 pregnant females, 9 breastfeeding females, and 43 health workers. Selection of new participants continued until research objectives were met and data saturation was achieved [38]. Mothers were partly selected during their visits to health facilities, when they received supplementary foods to treat malnutrition during pregnancy. Mothers were partly selected at home with the help of volunteer mothers. Religious leaders and traditional birth attendants were included because they enjoyed prestige in the community as experts able to explain community values, fasting rules, and diet diversity. Of religious leaders, only Muslim leaders were interviewed, because in the villages where the study was conducted, 99% of the population was Muslim. Most females were Muslim, but a few health workers were orthodox Christian and protestant. At the health centers, only staff active in malnutrition programs were involved in the research.

KD pretested the interview guides to assess the clarity and flow of the questions. On the basis of the pretest results, we adjusted the wording and sequence of the questions to ensure a natural flow of the conversation. The interview guides were tailored to specific study participants and data collection tools. One guide was used for pregnant females and breastfeeding mothers: another one for religious leaders and traditional birth attendants, and a third interview guide for health workers. The interview guides were adjusted for the focus group discussions. In-depth interviews included detailed questions, whereas the focus groups contained both specific and general questions. Each focus group discussion included between 7 and 10 participants and lasted 45–60 min.

The one-on-one in-depth interviews were conducted at health facilities, the mothers’ homes, and in community buildings, such as schools and village leaders’ offices. Although a bias toward more socially approved answers might be expected at the health centers, we did not find systematic differences in the answers of females interviewed at health centers and at home. Data collection was conducted in the local language, Afaan Oromo, to ensure that all participants could fully engage in the process. Participant observation was conducted at the health facilities and in the villages for a period of over 5 mo to see mothers’ actual dietary practices, which occasionally differed from their views expressed in interviews and focus groups. Systematic notes were taken of observations made at each visit to health facilities and the villages.

### Data analysis and interpretation

The interviews and focus group discussions were audio recorded and transcribed verbatim manually for coding and analysis. We analyzed and interpreted the data through an iterative process of data collection and analysis. We took 6 steps in the thematic analysis: familiarizing ourselves with the data; identifying initial codes; exploring themes; reviewing themes; defining and naming themes; and writing up [39]. Multiple readings of transcripts from interviews and focus group discussions were conducted, which were then analyzed using NVivo version 15 software for managing and coding large qualitative data sets. The data were coded using an inductive approach, finding consensus on these themes. We have used the Standards for Reporting Qualitative Research framework, depicting study context, findings, analysis, and interpretation [40]. After the coding was ready, codes with similar information were grouped into categories, and categories were grouped into broader themes of mothers’ dietary practices. Thematic analysis helped us to examine the data by identifying, analyzing, and reporting recurring patterns across a dataset [41]. The themes emerged from the data analysis and were not selected before analysis, to profit maximally from the open interviews and focus group questions. We maintained the trustworthiness of the data in 5 ways. After an interview was conducted, KD summarized the main responses to see whether all questions had been answered. We held discussions with the research team to evaluate the credibility of the collected data. We tailored the interview guides for each category of study participants. Data collection was conducted only when participants felt comfortable answering the questions. Researchers maintained reflexivity by avoiding prior assumptions, collecting data without bias, and presenting findings ethically.

Three broader themes and 9 subthemes were identified.

1. Dietary habits.
2. Barriers to adequate dietary practices (family income, the sale of food items, prioritizing family members, dietary self-restraint, Khat use, religious beliefs and fasting).
3. Facilitators to adequate dietary practices (religious leaders’ view, cooking demonstrations, and pregnancy conferences).

## Results

### Sociodemographic characteristics of the study participants

A total of 112 participants took part in the study. Of these, 94.6% of the participants were females, and the majority were between the ages of 21 and 30. More than half of them never attended formal education and the majority, 95.5% of the participants, were Muslim and married (94.6%) (Table 1).

**TABLE 1:** Sociodemographic characteristics of study participants (*n* = 112).

Sociodemographic characteristics	Number of participants (*n*)	Percentages (%)
Sex		
Female	106	94.6
Male	6	5.4
Age group		
>40	4	3.47
31–40	43	38.3
21–30	55	49.1
≤20	10	8.9
Educational status		
No formal education	57	50.8
Diploma	52	46.4
First degree	3	2.6
Religion		
Islam	107	95.5
Orthodox Christian	4	3.5
Protestant Christian	1	0.9
Marital status		
Married	106	94.6
Widowed	—	—
Unmarried	6	5.3

### Dietary habits

Access to a diverse diet was a challenge during pregnancy. A pregnant female said during a focus group discussion, “During pregnancy, the variety of food I have mainly consists of flatbread and porridge. When I have support from God, I buy potatoes, fruits and vegetables. I eat food from cereals like maize and sorghum.” Interviews with pregnant females confirmed that the staple food for females during pregnancy included sorghum, wheat, maize, or a mixture of cereals, which can be consumed in the form of flatbread or porridge. A pregnant female told: “Stew made from peas, split fava beans, lentils, chickpeas, onion, tomato, and cooking oil is common.” Health workers were concerned about such a monotonous diet that the females talked about, because it could lead to nutritional deficiencies.

Dietary diversity was also an issue during the postpartum period. During participant observation, we met a female who had had a cesarean section 4 day earlier at the health facility and had a new infant. Three years ago, she also had a cesarean section when she delivered twins (a boy and a girl). Because of her previous cesarean section, the health workers insisted that she should have another surgical delivery, expressing their concerns about prolonged labor. A health worker recommended that she ate meat, eggs, honey, liver, and milk to recover from pregnancy and delivery. Local people also sometimes recommended eating liver for females with anemia. She told us that the health worker had not only advised her what to eat, but also what to avoid: mango juice, Fanta, and grocery biscuits because of the sugar content. However, disregarding the good advice, she could only afford milk 3 times, and for the rest, her income allowed her to eat potatoes and flatbread made from cereals. The affordability and availability of food reduced her dietary diversity during the postpartum period.

The standard beverage was a coffee made from the leaves of the plant, boiled with milk. The coffee made from leaves was believed to stimulate breastmilk production. A female said: “I drink coffee made from coffee bean leaves regularly, and it increases the amount of breastmilk for my child.”

The boiling of tea and coffee was a good way to purify water, because the lack of clean water was a significant issue. One mother shared: “I walk for over two hours to fetch water.” This struggle was a daily reality in some villages, with mothers undertaking long journeys to ensure their families had access to water. Several mothers reported that unclean water could cause stomach pain and diarrhea in their children. The use of some chemicals was an alternative for purifying water. Health workers reported about nongovernmental organizations that, some time ago, had assisted families using clean water. Health workers recommended Wuha Agar, a chlorine-based water treatment solution. Health workers taught mothers how to use Wuha Agar by instructing them to add 1 capful of the solution to 20 L of water and let it sit for 30 min before use. However, the availability of Wuha Agar was limited during fieldwork. A few mothers mentioned that they treated water by boiling it. However, pregnant females themselves often drank water without boiling it. Water insecurity encumbered food preparation, because porridge and flatbread bread cannot be prepared without water.

### Barriers to adequate dietary practices

Several factors hindered females’ ability to maintain a diverse diet. These factors include inadequate family income, the sale of food items, the prioritization of other family members, beliefs about necessary dietary self-restraint, khat use, and religious beliefs and fasting.

### Inadequate family income

Lack of income negatively influenced a diverse diet. Mothers told us how a low family income prevented them from accessing fruits and meat, despite their desire to include them in their diet. One female recalled that *“*during my pregnancy, I had intense cravings for fruits and meat, but I could not afford to buy them.” KD observed how expensive meat and fruits were in local markets. Mothers prepared meals for the entire family rather than making special meals for their children, adjusted to their age. As 1 mother said, *“*Our younger children eat the same foods [as we do], and we cannot prepare separate meals due to financial resources when making porridge or flatbread with stew.” Others however were able to serve their children without significant extra costs. One mother of young children mentioned that children have fast digestive systems and need small portions of food every few hours. She prepared a significant portion of porridge in the morning and served it to them whenever they cried, at intervals of a few hours. Financial constraints often compelled mothers themselves to adopt the same diet. Pregnant females reported: “Porridge is served with cow milk for those who afford to buy milk. However, pregnant females who cannot afford cow milk use cooking oil.” Community mothers also said that: “those who cannot afford milk can use sugar and hot pepper with a mix of cooking oil (local sauce).” Inadequate family income thus prevented pregnant females from eating a diverse diet. It was observed that in June, the prevalence of malnutrition among mothers and children was higher than in March.

### The sale of food items

Some participants mentioned, and on multiple occasions, it was observed that, although eggs and milk were commonly available at home to enrich the diet of pregnant females and children, they frequently sold these items and, with the proceeds, bought other food that met the family’s needs. For instance, selling 2 or 3 eggs could allow a mother to purchase 1 or 2 kg of rice, which would meet the food needs of the entire family. Two or 3 eggs might have been cooked to enrich 1 child’s breakfast, but 1 kg of rice met 3 children’s and their parents’ food needs. As 1 female stated, “We have milk and eggs, but we often take them to the market to buy more food to feed our children.” Selling eggs and milk for purchasing other family foods increased quantity but reduced diversity.

Females also sold khat to purchase spaghetti or macaroni to support the family diet. Spaghetti and macaroni were practical because they were accessible and affordable. These food items are common in Eastern Ethiopia, and KD observed that, thanks to the proximity to Djibouti and Somalia, good quality spaghetti and macaroni could be imported at a lower price than locally grown cereals, especially when it was smuggled. Spaghetti and macaroni, made from wheat, are quite like porridge and flatbread in terms of their nutritional content. Another popular packed food of Western origin was biscuits, which are often unhealthy and do not fulfill the dietary requirements of children. Another mother confessed: “I have goats, potatoes, and onions, but I sell them and end up buying less nutritious food, which shows a lack of awareness.” She contradicted herself when she said she lacked awareness, but she was not alone in recognizing the importance of nutritious food and nevertheless practicing it infrequently. Health workers disagreed with these practices, selling nutritious foods, but teaching mothers to change this behavior has not been successful.

### Prioritizing other family members

Our findings show that usually the husband ate first, then the children, and finally the mothers, also when they were pregnant or breastfeeding, although sometimes husband and wife ate together. A female explained: “In our tradition, the husband is the head of the household. Wives serve their husbands first with a good meal.” They not only ate first but also received the best portion, and this norm was strong. One mother, for instance, feared community gossip: “I do not want to be called in the community as women who eat before her husband.” The gendered division of tasks in the household also became clear from the fact that bringing tea and lunch to a husband working at the farmland was widely considered an obligation for wives. As 1 mother said: “Bringing coffee and lunch is my responsibility unless my husband is angry at me.”

The preparation of meals was even contingent on the presence of the male heads of the households. Some females indicated that when their husbands were away, they tended to prepare fewer meals or meals of lower quality. One mother noted, “When my husband is not around, we often have smaller portions, and we cook more when he is home.” Another female asserted, “If my husband is not home, I skip breakfast and eat when he returns.” The norms of husbands eating first sometimes resulted in mothers and children receiving less food in quantity and quality.

### Dietary self-restraint

Most females believed they had to avoid certain foods during pregnancy to ensure their safety and minimize complications during delivery. One pregnant mother mentioned that: “Certain foods, such as cabbage, red root vegetables, bananas, and potatoes, can contribute to an increase in fetal size, which may lead to the necessity of a cesarean section during delivery.” Another female added: “Dairy products, eggs, and meat can also lead to excessive weight gain in their children during pregnancy, complicating the delivery process.” A third mother elaborated that there were not only concerns about their health: “Cesarean birth [also] increases the cost of care.” Health workers were aware of these beliefs among the females, but disagreed. One health worker reported: “Pregnant women often believe that eating potatoes and vegetables causes the child to grow big, leading to the need for a cesarean delivery. This is a misunderstanding among pregnant women.” As a solution to what the health workers saw as misconceptions, they emphasized the importance of educating pregnant females.

Nevertheless, the mothers were resolved; they had to show self-restraint and limit the consumption of vitamin- and protein-rich foods. Consequently, many mothers desired to include more flatbread in their diets, which contains less vitamins and protein. Their conviction arose from known cases of maternal deaths during delivery in their communities. The females perceived a clear link between food, the weight of the infant, and risk of death. As 1 mother put it “Consuming proteins and vitamins could result in severe complications during delivery, mainly if the infant’s weight is significant, which could even lead to maternal death.” Although they felt assured that medical care could be sought during prolonged labor and warning signals, they preferred to deliver naturally at home. This was an extra reason for them to be worried about infants with heavy weights.

Reasons for avoiding certain foods were not limited to the alleged impact on delivery but also pertained to stereotypes associated with certain foods. Food signaled the social class of families. Vegetables were often perceived as food for low-income people, whereas dairy products and meat were associated with wealth. To avoid the embarrassment of being labeled “poor” by society, some mothers chose to limit their consumption of cabbage. One mother said, “I prepare porridge, mix it with cabbage, and give it to my children, but people around me imply that cabbage is for the poor. I feed my children in secret.”

### Khat use

The tradition of chewing khat is a common practice in the region, and the KD observed that many mothers chewed khat during lunchtime, before meals, and while waiting at health facilities or in the marketplace. As 1 pregnant female explained: “I chew khat during lunchtime. It is a tradition, and I find it hard to stop even because of my pregnancy.” However, as other participants remarked, chewing khat reduced appetite and this was confirmed by a health worker: “Chewing khat during pregnancy reduces women’s appetite and negatively affects maternal health and unborn fetuses.”

### Religious beliefs on fasting

Most of the study participants were Muslim, and fasting is a common religious practice in Eastern Ethiopia. During Ramadan, Muslim mothers engaged in fasting, prayer, and spiritual reflection. Although fasting, Muslims abstained from eating or drinking anything during daylight hours, which meant they skipped meals. One pregnant female stated: “I must fast during fasting session. Everything is Allah’s will.” Another pregnant female said, “I do not want to stop fasting, as it is a spiritual practice and responsibility.” In contrast, still other female said: “I can refrain from fasting if I feel unwell and I compensate after delivery.” A Muslim religious leader confirmed, “Women are encouraged to fast, but if they feel unhealthy during pregnancy, they are permitted to stop fasting and can compensate for the missed fasts later.” Also, after delivery, Muslim religious leaders noted that: “for a period of 40 to 60 days, women may refrain from fasting but should make up for the missed days afterward.”

### Facilitators to adequate dietary practices

Religious leaders and health workers, making use of cooking presentations and pregnancy conferences, can have a positive impact on dietary diversity.

### Religious leaders’ nutritional knowledge

Religious leaders not only sanctioned pregnant females and new mothers to eat during fasting hours but also highlighted the significance of diverse dietary practices. A religious leader reported during interviews: “Camel milk, camel meat, and goat meat have medicinal benefits as foods.” He framed this opinion in religious terms: “Camels and goats consume medicinal plants, and religious teachings endorse these benefits.” Another religious leader said: “I encourage people to consume honey rather than selling it in the market.” Additionally, a religious leader emphasized the importance of temir fruit, which grows on the date palm tree, for young children and mothers. Garlic was also mentioned as an important part of a healthy diet by the religious leader: “Garlic has medicinal benefits for various illnesses.” Although these advice of religious leaders focused on alleged medicinal qualities of food, they did promote dietary diversity.

### Cooking demonstrations

Cooking demonstrations were participatory events that could promote dietary diversity. Participant observation helped to understand the aim and process of the event. The sessions included pregnant females, breastfeeding mothers, and mothers with children ≤2 year joined. Local health workers coordinated with village leaders or female volunteers to encourage mothers to attend. The demonstrations were scheduled every 3 months at health posts, where health workers explained the importance of dietary diversity, making use of local ingredients in daily food preparation. A female reported after a cooking demonstration: “Before, I did not have the opportunity to learn how to prepare nutritious porridge from a variety of cereals, including cabbage, and proteins. I have some items, but I only use maize or sorghum to prepare porridge for my child and myself during pregnancy.”

To make the session participatory, females were encouraged to bring ingredients to the demonstration, but it was not mandatory. On average, 30 females attended these meetings. Participants often shared their own experiences among themselves, discussing topics such as how they prepared porridge, their cravings during pregnancy, and their breastfeeding experiences. The cooking sessions included a mother’s consideration of the 6 food groups that are important for pregnant females and children (fruits, proteins, dairy products, vegetables, fats and oils, and carbohydrates). The interactive nature of cooking demonstrations allowed pregnant females to engage actively, ask for clarifications, and learn from each other, rather than receiving cooking instructions through lecturing.

### Pregnancy conferences

Pregnancy conferences formed another occasion where community health workers educated pregnant females on diet diversity during pregnancy, antenatal care, delivery at health centers, and vaccinations. On mean, 20 females attended these conferences. Health workers taught the females how to prepare porridge using various cereals, using 14 different ingredients, but 8–9 different ingredients were used at most during cooking demonstrations. Health workers also told pregnant females to add an extra meal to their diet. A pregnancy conference participant told*:* “I've learned that I should add one extra meal during pregnancy. If I eat twice before, I need to add one extra meal due to pregnancy.” The health workers also used the pregnancy conference to promote delivery at the health center. A health worker told: “Some pregnant women choose to give birth at home because they do not know exactly their pregnancy months. As a result, they may suddenly go into labor at home, and if the labor is not too intense, they end up delivering the baby there (at home).” The pregnancy conference was the sole event organized specifically for pregnant females, and this platform allowed health workers to create awareness about the importance of dietary diversity during pregnancy. Moreover, they formed a platform for pregnant females to learn from females who had already experienced pregnancy.

## Discussion

Dietary practices, including what, how, and when pregnant females eat, affect the health of the mother and the child. Adequate dietary practices before and during pregnancy are associated with a reduced risk of disorders of pregnancy, including gestational diabetes mellitus, preterm birth, obesity, preeclampsia, and gestational hypertension [5].

Cultural practices have a significant influence on food preferences [42]. Our research area was characterized by cereal-based meals, and for pregnant females, porridge and flatbreads were common staple foods. Literature indicates that such a diet can be monotonous, lack diversity, and is linked to deficiencies in micronutrients, negatively impacting both maternal and newborn health [43,44]. A systematic review of literature confirmed that a high intake of vegetables, fruits, whole grains, dairy products, and protein during pregnancy reduces risk of having a low birth weight [14]. Country-specific studies also demonstrated that a lack of a properly diversified diet is linked to a higher risk of being underweight in children aged 6–23 months, and detrimental effects on children’s physical and cognitive development [[45], [46], [47]].

Our study identified barriers and facilitators to dietary practices among pregnant and breastfeeding females. Inadequate family income, the sale of self-produced healthy food items to increase the quality at the expense of the quality of food, prioritization of other family members, dietary self-restraint, khat use during pregnancy, and religious beliefs and fasting were identified as barriers. The inequalities in power within a household affected the dietary intake of mothers. The common practice of husbands eating first, followed by children, and mothers last can lead to less quantity and quality of food for mothers and children. In Ethiopia, social norms regarding intrahousehold food allocation begin during adolescence. A previous study revealed that adolescent girls are expected to eat only after serving male family members [28]. Men are often considered the breadwinners in their families [48]. However, the differences between females and males in Eastern Ethiopia did not go as far as rural Kenya, where preferential food allocation prohibits the consumption of eggs, chicken, fish, and meat to females and children [49]. Self-imposed dietary restrictions during pregnancy were a significant challenge in meeting dietary needs. In some regions of Ethiopia, avoiding food items (meat, salt, egg, cabbage, green leafy vegetables, yogurt, cheese, sugar cane, and green pepper and milk) is linked to fear of preeclampsia, fetal deformities, plastered on the fetal head, fear of abortion, evil eye, leaf passes to the womb and attaches to the infant’s head [50,51]. Such themes did not emerge within the current study. However, pregnant and breastfeeding females held a strong conviction that consuming protein, vitamins, and dairy products during pregnancy could lead to excessive weight gain in children, potentially complicating delivery and possibly resulting in a cesarean section and even the death of the mother. These findings align with other studies in Ethiopia [32,33]. It was on this point that health workers, who were aware of the self-imposed restrictions of the pregnant females, strongly disagreed with the females. They encouraged mothers to consume protein- and vitamin-rich foods throughout pregnancy. However, they have had little success in changing these conceptions. Health workers reported that implementing sociobehavioral changes in dietary practices required continuous health education efforts, and they attributed their limited success to unforeseen irregularities in planned extension activities.

Khat use was found to be a barrier to dietary diversity during pregnancy. Consistent with previous research, chewing khat could reduce the appetite of pregnant mothers, leading to decreased food consumption and nutrient intake, which in turn may negatively impact fetal growth and overall health [52]. Maternal psychological stress and anemia have been reported among females who chewed khat during pregnancy [53], and khat chewing in pregnancy has also been associated with prelabor rupture of membranes in similar sites [54]. Religious beliefs and fasting during pregnancy formed another element of dietary practices. Islam was the predominant religion in the study area, and it was common to fast during Ramadan. Although females said that Islamic rules allowed pregnant females to postpone fasting, and compensate for the missed days after giving birth, some pregnant females refused to eat and drink during fasting hours. The females did not eat during the daytime, but did not abstain from eating animal and dairy products, as is expected from followers of the Orthodox Tewahedo Church, the second religion in Eastern Ethiopia, during the Christian fasting [32,33]. In contrast to other Christian parts of the country, where locally made alcoholic beverages are acceptable during pregnancy [32,33,55], alcoholic drinks were, because of Islam, forbidden in our study area.

The positive roles of religious leaders, cooking demonstrations, and pregnancy conferences as health forums facilitated dietary practices among pregnant females. In the current study, religious leaders supported pregnant females consuming nutritious foods, including camel milk, camel meat, and goat meat, for their medicinal benefits. Additionally, they highlighted the importance of temri fruit and garlic. Religious leaders emphasized that pregnant females could postpone fasting and make it up later, which is a positive aspect of dietary diversity. In contrast, in other parts of Ethiopia, religious leaders only allow pregnant females to eat breakfast except milk, animal-source foods, and meat during fasting periods [32].

Cooking demonstration sessions were another facilitator for dietary practices. Community health workers organized these sessions to teach pregnant females, breastfeeding mothers, and mothers with children under 5 year old about the importance of dietary diversity and the use of local varieties of cereals, vegetables, fruits, vitamins, and proteins to prepare nutritious porridge for pregnant females and their children. In these sessions, health workers encouraged mothers to consider the 6 food groups that are important for pregnant females and children (fruits, proteins, dairy products, vegetables, fats and oils, and carbohydrates). Additionally, this session allowed pregnant females to learn from each other about how they prepared porridge, their cravings during pregnancy, and their breastfeeding experiences. Though these sessions were helpful, their impact was limited by the irregular frequency of sessions and the limited variety of food at home to implement the lessons learned. Cooking demonstration sessions are a common practice to teach dietary diversity in Ethiopia [56].

Pregnancy conferences were the final facilitators in promoting dietary diversity. They were another occasion when community health workers educated pregnant females on dietary diversity during pregnancy, antenatal care services, delivery at health centers, and the importance of vaccinations for pregnant females and their children. The pregnancy conferences were the sole events organized explicitly for pregnant females. Pregnant females who attended these sessions learned from each other, and they were particularly a good opportunity for newly pregnant females to learn from females who had already been pregnant before. These sessions were also affected by irregularities and low attendance of pregnant females due to the distance from home to the health facilities and household responsibilities to attend the sessions. Institutional delivery among pregnant females who attended this session is higher than among those who did not [57].

However, when we contextualized dietary practices in wider social processes, two societal frictions became clear that could hamper an improvement of dietary diversity. First, changing a hegemonic culture where husbands ate before their wives and received larger and better portions requires a change in females and males. A different order of consuming food is more than a nutritional change and would come down to a fundamental change in gender relationships in the household. Although few males are likely to object to adequate nutrition for their wives and children, changing the gender balance will evoke more resistance.

Second, the self-imposed restriction on food by pregnant females was the issue on which pregnant females and health workers disagreed most. The health workers could focus on adequate nutrition, and neglect of their sound advice was easily perceived by them as a misconception on the side of the females. The females, in contrast, had more priorities to think of. Fears for a cesarean section or maternal death at delivery, reluctance to move from home to a health center for delivery, and concerns about gossiping about class status were all founded on their own experiences and concrete stories from and about other females. Such concerns of the mothers should be taken into consideration by the health workers. It is telling in this context that the cooking demonstrations and pregnancy conferences were relatively successful in conveying information about healthy dietary practices to the mothers; these were events where the females could actively participate and exchange their views. This study suggests that health workers had the most success with nutritional interventions when they took the input of the females seriously.

### Limitations of the study

The strength of this study lies in the diverse perspectives on dietary practices from pregnant and breastfeeding females, religious leaders, traditional birth attendants, and health workers. Additionally, participant observations yielded findings that complemented the interview and focus group discussions. However, the lack of insight into husbands’ views on one important aspect of dietary practices, the unequal allocation of food within households, was a limitation. Future studies should consider male perspectives on gender balance in households in general, and the relationship between eating order at home and female health in particular.

In conclusion, the study found that social and cultural barriers and facilitators influenced dietary practices in Eastern Ethiopia. Inadequate family income, the sale of self-produced food to purchase larger quantities of less diverse food, prioritizing husbands in the allocation of food in the household, self-imposed restrictions to prevent a fetus from growing too big out of fear for prolonged labor, a cesarean birth and maternal death, khat use, and religious fasting were identified as barriers. The religious leaders’ opinions in promoting particular food, successful cooking demonstrations, and pregnancy conferences were facilitators of healthy dietary practices. Understanding dietary practices can help health workers develop effective nutritional interventions for pregnant females, thereby improving maternal and child health. However, the females had partly different priorities than the health workers in making dietary choices and kept more factors in mind than nutritional considerations. Cooking demonstrations and pregnancy conferences were effective moments to encourage pregnant females to increase dietary diversity, because there the females had the opportunity to exchange experiences. Future study is necessary on the gender roles of wives and husbands and in particular conditions where equal rights to food in families might be observed. Furthermore, research is necessary under what conditions health workers and females might find a balance between the importance of healthy food and the mothers’ worries.

## Author contributions

The authors’ responsibilities were as follows – KD, LS, FC, KTR: designed the study; KD: recruited the study participants, collected the data, transcribed the interviews and focus group discussions, and analyzed the data. KD, LS, FC, and KTR discussed the various steps of fieldwork and analysis. KD: wrote the draft of the manuscript; and all authors: read and approved the final manuscript.

## Data availability

The data supporting the findings of this study are available on request from the corresponding author. The data are not publicly available due to containing sensitive information that could compromise the privacy of research participants.

## Funding

The BMGF supported this work through a data-to-action research grant.

## Conflict of interest

KD reports financial support was provided by Bill & Melinda Gates Foundation. All other authors report no conflicts of interest.
